# MET and PI3K/mTOR as a Potential Combinatorial Therapeutic Target in Malignant Pleural Mesothelioma

**DOI:** 10.1371/journal.pone.0105919

**Published:** 2014-09-15

**Authors:** Rajani Kanteti, Immanuel Dhanasingh, Ichiro Kawada, Frances E. Lennon, Qudsia Arif, Raphael Bueno, Rifat Hasina, Aliya N. Husain, Wickii Vigneswaran, Tanguy Seiwert, Hedy L. Kindler, Ravi Salgia

**Affiliations:** 1 Department of Hematology/Oncology, University of Chicago, Chicago, Illinois, United States of America; 2 Department of Pathology, University of Chicago, Chicago, Illinois, United States of America; 3 Department of Surgery, Brigham and Women's Hospital, Harvard Medical School, Boston, Massachusetts, United States of America; 4 Department of Surgery, University of Chicago, Chicago, Illinois, United States of America; Cedars-Sinai Medical Center, United States of America

## Abstract

Malignant pleural mesothelioma (MPM) is an aggressive disease with a poor prognosis. Studies have shown that both MET and its key downstream intracellular signaling partners, PI3K and mTOR, are overexpressed in MPM. Here we determined the combinatorial therapeutic efficacy of a new generation small molecule inhibitor of MET, ARQ 197, and dual PI3K/mTOR inhibitors NVP-BEZ235 and GDC-0980 in mesothelioma cell and mouse xenograft models. Cell viability results show that mesothelioma cell lines were sensitive to ARQ 197, NVP-BEZ235 and GDC-0980 inhibitors. The combined use of ARQ 197 with either NVP-BEZ235 or GDC-0980, was synergistic (*CI*<1). Significant delay in wound healing was observed with ARQ 197 (p<0.001) with no added advantage of combining it with either NVP-BEZ235 or GDC-0980. ARQ 197 alone mainly induced apoptosis (20±2.36%) that was preceded by suppression of MAPK activity, while all the three suppressed cell cycle progression. Both GDC-0980 and NVP-BEZ235 strongly inhibited activities of PI3K and mTOR as evidenced from the phosphorylation status of AKT and S6 kinase. The above observation was further substantiated by the finding that a majority of the MPM archival samples tested revealed highly active AKT. While the single use of ARQ 197 and GDC-0980 inhibited significantly the growth of MPM xenografts (p<0.05, p<0.001 respectively) in mice, the combination of the above two drugs was highly synergistic (p<0.001). Our results suggest that the combined use of ARQ 197/NVP-BEZ235 and ARQ 197/GDC-0980 is far more effective than the use of the drugs singly in suppressing MPM tumor growth and motility and therefore merit further translational studies.

## Introduction

Malignant pleural mesothelioma (MPM) is an aggressive tumor originating in the serosal surfaces of the pleura, peritoneum, pericardium, and tunica vaginalis [1]. MPM is diagnosed in about 3500 Americans each year; and its incidence is still increasing in many areas of the world [2]. While the principal etiological factor is exposure to asbestos, genetic predisposition, prior exposure to Simian Virus 40 (SV40) and radiotherapy can trigger the development of MPM [3]–[5]. The standard therapy for MPM is combination chemotherapy with cisplatin and an anti-folate analog [6], [7]. Despite recent advances, this disease has a poor prognosis and the median survival time is about one year [8], clearly there is an urgent need for more efficacious therapeutics.

Receptor tyrosine kinases (RTKs) play a crucial role in tumor growth and metastasis, providing key signals that lead to transformation, proliferation and invasion [9]. Various studies have shown that RTKs including epidermal growth factor receptor (EGFR), MET, insulin growth factor receptor (IGFR) and vascular endothelial growth factor receptor (VEGFR) are expressed in MPM [10]–[14]. HGF (hepatocyte growth factor)/MET signaling pathway is associated with acquired resistance to EGFR inhibitors in EGFR mutant non–small cell lung cancers [15]. It is therefore important to target MET along with a complementary target that can potentially synergize to kill cancer cells and ward off resistance.

We previously reported that MET is overexpressed and mutated in a variety of malignancies including MPM [13]. Using several mesothelioma cell lines, our group showed that the small molecule MET inhibitor SU11274 suppresses cell proliferation. ARQ 197 (Tivantinib) is a non-ATP competitive inhibitor of MET that binds to the non-phosphorylated inactive form of MET. Preclinical studies show that ARQ 197 inhibits MET activation in multiple cancer cell lines [16]. In this study, we determined the efficacy of ARQ 197 in suppressing the growth of MPM cells and tumors.

A key downstream signaling molecule for MET and other RTKs is phosphatidylinositol 3′ kinase (PI3K), a cellular oncogene and essential intracellular lipid kinase [17]. The p110α catalytic subunit of PI3K and its constitutively bound regulatory subunit p85 are usually overexpressed and acquire frequent gain-of-function mutations in MPM. The phosphatidyl-inositol-3,4,5-trisphosphate (PIP3) generated by the PI3K at the cell membrane recruits PH domain containing proteins such as PDK1 and AKT to the plasma membrane resulting in activation of mTOR complexes. The AKT and mTOR signaling cascades promote cell proliferation and tumorigenesis. Therefore it is more effective to simultaneously target both mTOR and PI3K. Several inhibitors that target either PI3K alone or PI3K/mTOR are currently in phase I cancer clinical trials [18].

GDC-0980 and NVP-BEZ235 are potent, orally bioavailable, new generation small molecule dual inhibitors of class I isoforms of PI3K and mTOR. Studies have shown that both NVP-BEZ235 and GDC-0980 significantly inhibit PI3K and mTOR activity, and tumor growth in many preclinical cancer models. NVP-BEZ235 and GDC-0980 are currently in phase I clinical trials in patients with solid tumors [19]–[23].

Here we have determined the combinatorial efficacy of MET inhibitor ARQ 197 and dual inhibitors of PI3K/mTOR in MPM. Our studies clearly demonstrate that the combined use of ARQ 197 with GDC-0980 or NVP-BEZ235 results in significant synergy in suppressing MPM cell proliferation and tumor growth.

## Materials and Methods

### Antibodies and Reagents

Antibodies for p110α, p-85, AKT, p-AKT ^Thr308^, p-AKT ^ser473^, S6, p-S6 ^Ser235/236^, cleaved PARP, total PARP, cyclin D1, p-MET (1234/1235) and anti-MAPK antibodies (ERK and p-ERK) were from Cell Signaling (Danvers, MA). Antibodies against total Met, p-MET (pY1349 and pY1003) and Alexa Fluor Phalloidin 594 were from Invitrogen (Grand Island, NY). PIP3 antibody was from MBL Co. Ltd (Japan). β-actin antibody was from Sigma (St. Louis, MO).

Recombinant human HGF was from R&D Systems (Minneapolis, MN). Wortmannin and LY294002 were from Cell Signaling. Crizotinib, GDC-0941, GDC-0980, ARQ 197 and NVP-BEZ235 were purchased from Selleck (Houston, TX). Stock solutions were prepared in DMSO and stored at −20°C till further use.

### Cell Lines

Seven human mesothelioma cell lines (H2596, H513, H2461, H2052, H2452, H28 and H2373) and one nonmalignant transformed mesothelioma control cell line (Met-5A) were obtained from American Type Culture Collection (ATCC) (Manassas, VA). All were cultured in RPMI 1640 medium (Gibco/BRL) supplemented with 10% (v/v) fetal bovine serum (FBS), L-glutamine and 1% penicillin-streptomycin at 37°C with 5% CO_2_. Met-5A cells were cultured in M199 media supplemented with various growth factors according to manufacturer's instructions (ATCC).

### Cell Lysis and Immunoblotting

Cells were plated in 10 cm dishes in 10 ml RPMI and incubated at 37°C. They were treated with indicated concentration of ARQ 197, NVP-BEZ235, GDC-0980 and combination of ARQ 197/NVP-BEZ235 and ARQ 197/GDC-0980 for the time indicated. Following treatment, whole cell lysates were prepared and proteins were detected by immunoblotting as previously described [24].

### Cell Viability

Exponentially growing cells were plated in 96 well flat bottom plates in 10% FBS media for overnight. Next day they were treated with the indicated drugs for 72 h. For each treatment eight wells were used. The viability of cells was measured using Alamar Blue method as described previously [24]. Values were normalized to untreated controls to generate dose response curves. Each experiment was repeated at least 3 times. IC_50_ values were generated for all the cell lines using ‘Prism’ software.

### Apoptosis Assay

H2596 cells were plated in 60 mm tissue culture plates overnight. Next day the cells were treated with the indicated drugs. After 48 h treatment, apoptosis was evaluated using Annexin V apoptosis kit from BD Biosciences (San Jose, CA) as per the manufacturer's protocol.

### Cell Cycle Analysis

H2596 cells were plated in 60 mm tissue culture plates overnight. Next day the cells were treated with the indicated drugs for 48 h. Cells were fixed in 70% ethanol in PBS, and stored at −20°C for 24 h. Next day the cells were washed with cold PBS twice and then resuspended in 300 µl of PBS containing RNase A and incubated at 37°C for 1 h followed by the addition of propidium iodide. Samples were analyzed by LSRII flow cytometer (BD Bioscience). The percentage of cells in different phases of the cell cycle was calculated by FloJo 9.3.0 software (Tree Star Inc., Ashland, OR).

### Tissue Microarrays (TMAs)

TMAs were assembled at the Brigham and Women's hospital at Boston, MA. The TMA had total 213 tumor samples, 196 normal lung samples and 14 control samples. A minimum of three tissue cores with a diameter of 1 mm was arrayed into a recipient block using an automated tissue microarrayer ATA-27 (Beecher Instruments, Sun Prairie, WI). All tissues were obtained under protocols approved by applicable IRBs and they were used for PTEN, and p-AKT staining. Tumor tissue immunohistochemistry (IHC) staining was performed using standard techniques as described previously [25]. All slides were reviewed by two independent pathologists who were blinded to the identity of the tissues. Two measurements, the percent and intensity of IHC staining were used to evaluate the level of protein expression in a tissue sample. The final IHC score was obtained by a semiquantitative method that accounts for staining intensity and percentage of cells stained. Scoring of the immunostaining in the nuclei was performed as follows: 0, no staining; 1+, weak staining; 2+, moderate staining; and 3+, strong staining.

For IHC staining the p-AKT ^Ser473^ antibody (Cell signaling, cat. # 4060) was diluted 1–20.

### Mouse Histology and Immunohistochemistry

Paraffin embedded blocks of all tumor samples were cut at 5 µM and each sample was stained with Hematoxylin and Eosin (H&E) for histologic analysis. IHC was done using CD31, MET, p-MET and p-AKT antibodies as described previously [25]. All samples were analyzed by scoring staining intensity.

### Dot Blot Assay

Dot blot assay for PIP3 was performed as previously described [26]. Briefly, cell lysates were prepared as described above and 50 µg was spotted directly onto a nitrocellulose membrane. The membrane was blocked in 5% BSA in 0.05% Tween/TBS for one hour at RT. The membrane was then incubated with the primary PIP3 antibody (MBL Co. Ltd, Japan) at 1 µg/ml in BSA TBS/T overnight at 4°C. The following day the membrane was washed and incubated with mouse secondary HRP-labeled antibody and proteins were visualized using an enhanced chemiluminescence reagent (BIORAD, Hercules, CA). Densitometric analysis was performed on the resulting images using Image J software (NIH, Bethesda, MA).

### Drug Synergy Assays

The cells were treated with a single drug and also with a combination of two drugs for 72 h. The synergy assay was done in two steps. In step 1, non-constant ratio of combination of two drugs was calculated by feeding the dose and effect data into CompuSyn to calculate the combinational index (*CI*). In step 2, the optimum ratio was selected from *CI* values, which is less than one and drug concentration less than IC_50_ for both the drugs. Using constant ratio, five different dose combinations of drugs were tested. The dose and effect data was entered into CompuSyn and synergy between the two drugs was determined. The analysis of synergy assay was done by the isobologram and combination- index methods, derived from the median-effect principle of Chou and Talalay using CompuSyn software (ComboSyn Inc.) [27].

### Wound Healing Assay

Cells (7×10^5^) were plated in 10 cm tissue culture plates overnight. Next day the cells were treated with the indicated drugs for 24 h. They were then trypsinized and replated in 24 well tissue culture plates containing cell culture inserts (Ibidi, Verona, WI). Next day the inserts were removed and the cells were washed with PBS and the media was replaced. The fine scratch created by the inserts was photographed at various time points and analyzed by TScratch software (CSELab, ETH Zurich, Switzerland).

### PamGene Assay

We used PamGene microarray technology (PamGene, Netherlands) to determine the activation status of various kinases. This assay measures specific peptide phosphorylation by protein kinases. The microarrays are embedded with 144 kinase-specific peptide substrates per microarray, which allows multiplex measurements. Fluorescently labeled anti-phospho-antibodies are used to detect phosphorylation. The protocol was followed as per manufacturer's instructions. H513 cells were treated with indicated concentrations of ARQ 197 for 4 h and the lysates were prepared as described above.

### Xenograft Mouse Tumor Model for ARQ 197 and GDC-0980

Female homozygous athymic nude mice aged 5-6 weeks from Harlan Laboratories (Indianapolis, IN). Animal care was in accordance with the Institutional animal care guidelines. 2.0×10^6^ H2596 mesothelioma cells were injected subcutaneously in the right flank of each mouse. Tumor growth was measured with calipers and volume (mm^3^) calculated as (L × W × H)/2. When the volume reached a mean of 200 mm^3^, mice were randomized into four groups (n = 10 mice/group) to receive vehicle alone, ARQ 197 alone (200 mg/kg), GDC-0980 (5 mg/kg) alone and a combination of ARQ 197 and GDC-0980. Drugs were administered once a day for 4 weeks by oral gavage. Body weight and tumor volume were recorded every 3 days until the study was terminated. Mice were sacrificed and tumor tissues were excised and fixed in 10% buffered formalin and embedded in paraffin.

### Ethics Statement

The female homozygous athymic nude mice (5–6 weeks age) were obtained and cared for according to institutional guidelines under a protocol approved by the University of Chicago Institutional Animal Care and Use Committee (Protocol number ACUP 72035). The Human TMA samples were obtained under The University of Chicago IRB protocol number 13473A-CR004 and Dana Farber Cancer Institute, Boston IRB protocol number 980-63. Tissue samples were obtained after informed consents were signed.

### Statistical Analysis

Statistical analysis was performed using GraphPad Prism version 5.0 (GraphPad Inc, San Diego, CA). In order to evaluate statistically significant differences between two continuous variables the unpaired Student's *t*-test was used. Differences were considered significant when *p*≤0.05.

## Results

### Expression of p-AKT and PTEN in Mesothelioma TMAs

**Figure 1:**
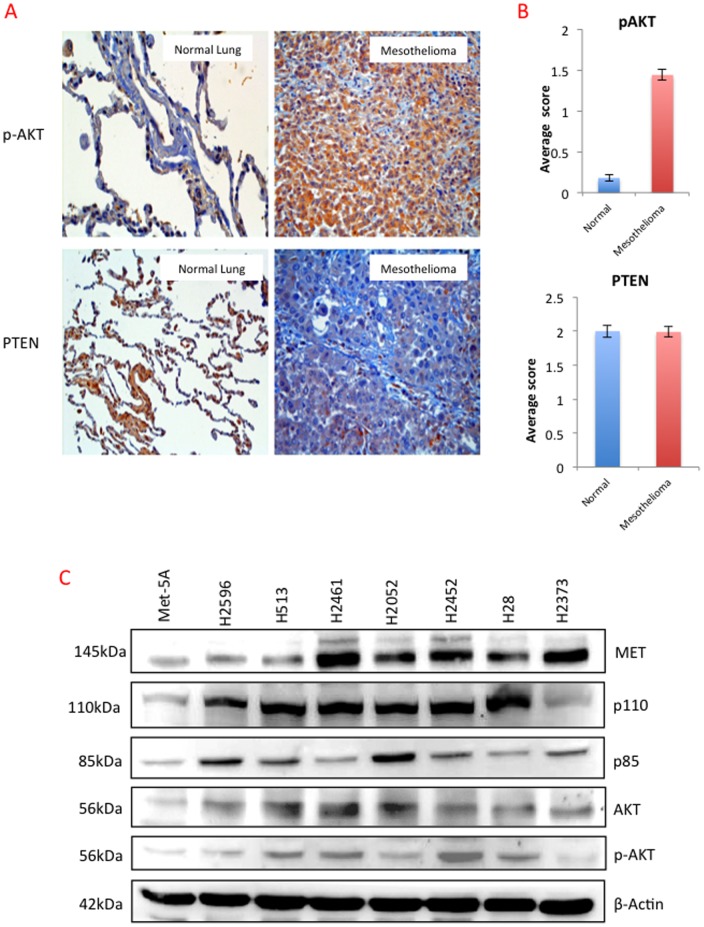
Expression of p-AKT and PTEN in archival mesothelioma tumor tissue samples. Mesothelioma TMA containing 212 tumor samples and 192 normal lung tissue samples probed with p-AKT and PTEN representative images are shown in **(A)**. **(B)** Comparative average scores of p-AKT and PTEN in TMA samples. **(C)** Representative immunoblot of mesothelioma cell lines for MET, p-110, p-85, Total AKT, p-AKT and loading control β Actin.

In order to discern the activity of PI3K in MPM tumors, we determined the phosphorylation status of its key substrate AKT and the expression levels of its negative regulator PTEN in archival mesothelioma tumor samples and their normal counterparts. A TMA of 213 MPM and 196 normal lung samples was generated and IHC was performed. The expression of p-AKT was significantly greater in tumor tissues than in matched normal tissue, however there was no discernible difference in PTEN expression (**Fig. 1A, B**). The above results suggested the presence of highly active PI3K in MPM tumor tissues.

### Expression of MET and PI3K/AKT in Mesothelioma Cell Lines

Using a panel of seven MPM cell lines and Met-5A as control, we determined the relative protein expression of p110α, p85, total AKT, p-AKT and MET using whole cell lysates and immunoblotting. As shown in **Fig. 1C**, all of the mesothelioma cell lines tested expressed varying but relatively higher levels of MET compared to Met-5A. All the MPM cell lines expressed significant but varying levels of PI3K (p85 and p110α) and AKT. H2373 and the control cell line Met-5A expressed lower levels of AKT in comparison to the other MPM cell lines examined. Taken together, both MET and PI3K are expressed at increased levels in the majority of MPM cell lines.

### MET and PI3K/mTOR Inhibitors Significantly Suppress MPM Cell Proliferation

**Figure 2:**
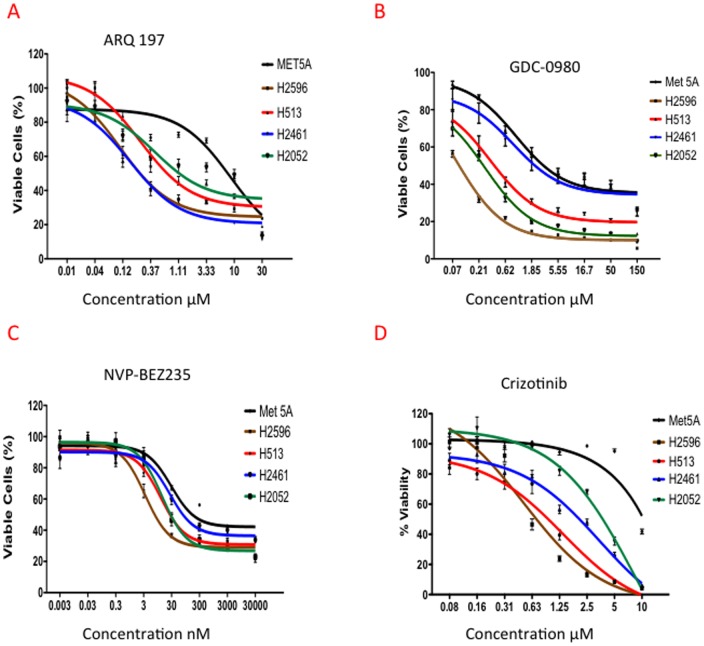
Effect of MET and PI3K/mTOR dual inhibitors on growth of human mesothelioma tumor cell lines. Met-5A, H2596, H513, H2461 and H2052 were treated with ARQ 197, NVP-BEZ235, and GDC-0980 for 72 h at the indicated concentrations. Viability was measured by Alamar Blue assay. The data shown represent the mean ± SEM. **(A)** ARQ 197, **(B)** GDC-0980, **(C)** NVP-BEZ235, **(D)** Crizotinib.

Cell viability was determined in MPM and the Met-5A control cells following treatment with increasing concentrations of ARQ 197, NVP-BEZ235 and GDC-0980 for 72 h. As shown in **Fig.** **2A**, cell viability was significantly decreased in all the MPM cell lines in response to ARQ 197 but not in Met-5A. The IC_50_s of ARQ 197 in MPM cell lines ranged from 0.1 to 0.4 µM in comparison, the IC_50_ for Met-5A was approximately 8 µM. We also tested the effect of crizotinib (**Fig 2D**), and the IC_50_ values were slightly higher in all the MPM cell lines tested compared to ARQ 197 (**Table 1**).

**Table 1:** IC_50_ of Mesothelioma cell lines with MET and PI3K/mTOR inhibitors.

	ARQ 197	NVP-BEZ235	GDC-0941	GDC-0980	Crizotinib
Cell Line	MET inhibitor	PI3K/mTOR inhibitor	PI3K inhibitor	PI3K/mTOR inhibitor	MET/ALK inhibitor
Met-5A	8.84 µM	29.39 nM	No Effect	0.96 µM	No Effect
H2596	0.11 µM	3.39 nM	0.66 µM	0.079 µM	0.48 µM
H513	0.25 µM	12.1 nM	0.62 µM	0.33 µM	0.47 µM
H2052	0.43 µM	13.86 nM	045 µM	0.27 µM	1.16 µM
H2461	0.16 µM	25.83 nM	1.35 µM	0.85 µM	2.91 µM

In the case of the PI3K/mTOR dual inhibitor GDC-0980, the Met-5A cells demonstrated higher levels of cell viability (IC_50_ 0.96 µM) in comparison to three out of the four MPM cell lines tested. The IC_50_s for H2596, H2052 and H513 ranged from 0.08 to 0.3 µM. In comparison, H2461 (IC_50_ 0.85 µM) behaved more like the control cells (**Fig. 2B**).

On the other hand, all the five cell lines were sensitive to NVP-BEZ235 and the IC_50_s ranged from 0.003 to 0.030 µM, with Met-5A being the least sensitive (**Fig. 2C**). **Table 1** is a summary of IC_50_s values for all the treatments. We then determined the efficacy of the combination of MET and PI3K/mTOR inhibitors and limited ourselves to two MPM cell lines, H2596 and H513.

### Effect of Combined Use of MET and PI3K/mTOR Dual Inhibitors on Cell Viability

**Figure 3:**
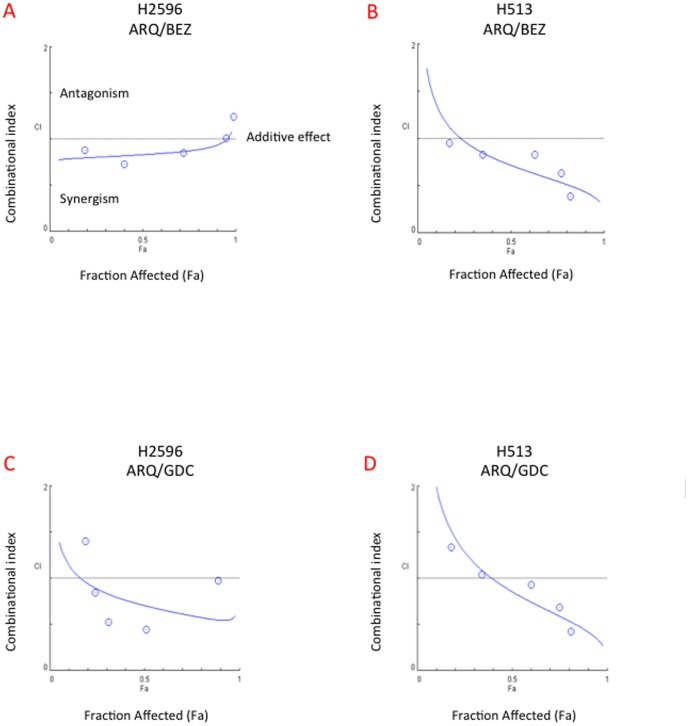
Synergistic anti-tumor activity of combination of ARQ 197 with NVP-BEZ235 and GDC-0980 in mesothelioma cell lines. Cells were treated with ARQ 197, NVP-BEZ235 and GDC-0980 alone or in combination at serial concentrations for 72 h. Cell viability was measured by Alamar Blue assay. Combination index (*CI*) plot analysis of ARQ 197/GDC-0980 and ARQ 197/NVP-BEZ235 combinations show that they interact synergistically in H2596 and H513 cells. *CI = 1* shows additive effect, *CI<1* is synergism and *CI>1* is antagonism. Each experiment was carried out independently and repeated at least three times. **(A)** *CI* plot of ARQ 197/BEZ235 in H2596 cells. **(B)** *CI* plot of ARQ 197/BEZ235 in H513 cells. **(C)** *CI* plot of ARQ 197/GDC0980 in H2596 cells. **(D)** *CI* plot of ARQ 197/GDC0980 in H513 cells.

H2596 and H513 were treated with a combination of ARQ 197/GDC-0980 or NVP-BEZ235 for 72 h and the viability was determined. The combination of ARQ 197/NVP-BEZ 235 had significant synergistic effect on the suppression of the growth of H513. The combination of ARQ 197/GDC-0980 on the other hand showed greater synergistic effect in H2596 compared to H513 cells in inhibiting cell growth. The synergy was calculated using combination index plots. (**Fig. 3A–D**). Since MET signaling is known to play an important and essential role in cell motility and metastasis [28]; we next determined the effects of these drug combinations on MPM cell motility.

### Effect of Combined Use of MET and PI3K/mTOR Dual Inhibitors on Cell Motility

**Figure 4:**
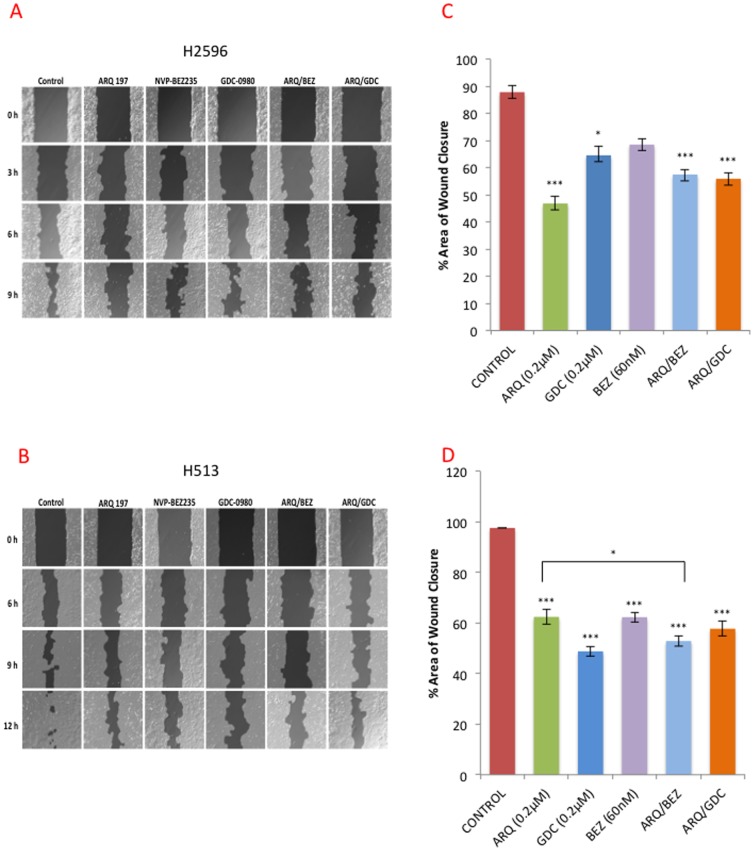
MET inhibition alone or in combination with PI3K/mTOR dual inhibitors suppresses cell motility. Wound healing assay was performed in H513 and H2596 cells treated with ARQ 197(0.2 µM), GDC-0980 (0.2 µM), NVP-BEZ235 (60 nM) or combinations for 24 h as described in the methods. Representative pictures of the degree of wound closure in control and treated **(A)** H513 and **(B)** H2596 cells at 12 h respectively. The open wound at each time point was quantified and normalized to 0 h for **(C)** H513 and **(D)** H2596 cells. The experiments were repeated three times in triplicate and average closure ± SEM is shown.

The H2596 and H513 cells were treated with the indicated drugs and their combinations for 24 h and scratch assay was performed. Wound closure was documented over a period of 12 h as shown (**Fig. 4A, B**). The open wound at each time point was quantified and normalized to 0 h value (**Fig. 4C, D**). In both H2596 and H513 cells, treatment with ARQ 197 alone was sufficient to significantly suppress cell motility and delay wound healing and there was no significant advantage to adding GDC-0980 and NVP-BEZ235. This supports the notion that MET signaling (independent of PI3K/mTOR) is a key contributor to MPM cell motility [13].

### Effect of Combined Use of MET and PI3K/mTOR Dual Inhibitors on Cell Cycle Arrest

**Figure 5:**
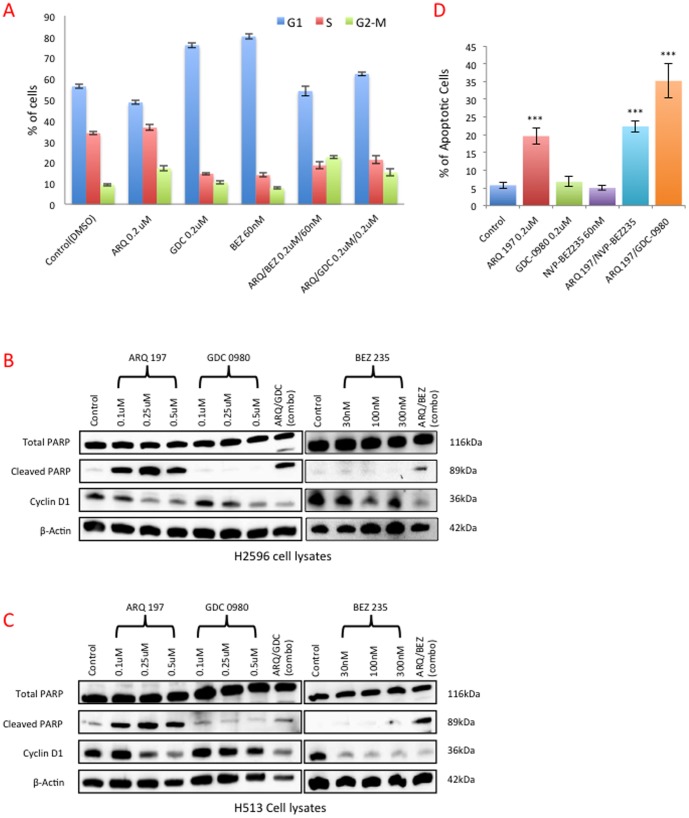
MET inhibition alone or in combination with PI3K/mTOR dual inhibitors induces cell cycle arrest and apoptosis. H2596 cells were treated with ARQ 197, GDC-0980, NVP-BEZ235 alone and in combination for 48 h. Cell cycle profile was determined using flow cytometry after staining with PI/RNase. **Fig.** **A** shows the percentage of cells in G1, S, and G2/M phases was quantified and the results expressed as the mean ± SEM of four independent experiments. H2596 **(B)** and H513 **(C)** cells, were treated with ARQ 197, GDC-0980, NVP-BEZ235 alone and in combination for 48 h. Cell lysates were prepared and immunoblotted for total PARP, cleaved PARP, cyclin D1 and actin as a loading control. H2596 cells treated with ARQ 197, GDC-0980, NVP-BEZ235 alone and in combination for 48 h, the cells were then stained with Annexin V-FITC/PI and analyzed by flow cytometry. Results are expressed as mean percentage of apoptotic cells ± SEM of four independent experiments **(D)**.

To investigate the underlying mechanisms related to the loss of viability and motility in MPM cells treated with MET and PI3K/mTOR inhibitors, we determined the effect of these inhibitors on cell cycle progression. H2596 cells were treated with the indicated inhibitors for 48 h, and cell cycle phases were analyzed by flow cytometry. Treatment of H2596 cells with either of the PI3K/mTOR inhibitors, GDC-0980 or NVP-BEZ235 for 48 h significantly arrested the MPM cells in G0/G1 phase as compared to the control group. On the contrary, treatment with ARQ 197 arrested the cells in G2/M phase. Interestingly, the combination arrested the cells in G2/M but not G1 phase (**Fig. 5A** **and Fig. S1A, B,**).

### Effect of Combined Use of MET and PI3K/mTOR Inhibitors on Apoptosis

Since growth arrest of cells, especially at G2/M phase can trigger apoptosis [29], we next determined the levels of cyclin D1 and cleaved-poly (ADP-ribose) polymerase (PARP) in the treated cells. The levels of cyclin D1, which is a G0/G1 cell cycle regulator [30] were decreased in cells treated with ARQ 197, GDC-0980 or NVP-BEZ235, in a dose dependent manner. In the case of NVP-BEZ235 the effect was much more pronounced in H513 cells compared to H2596 (**Fig. 5B, C**). However, the combination of ARQ 197/GDC-0980 and ARQ 197/NVP-BEZ235 induced the largest decrease in cyclin D1.

The combinatorial treatment of ARQ 197/GDC-0980 and ARQ 197/NVP-BEZ235 however induced significant levels of cleaved PARP in both MPM cell lines. Individual treatment with GDC-0980 or NVP-BEZ235 had little effect as evidenced from cleaved PARP levels (**Fig. 5B, C**). Similar results were obtained via immunofluorescence staining of cleaved PARP (**Fig. S2**). However ARQ 197 alone was extremely effective in the induction of cleaved PARP in both MPM cell types, suggesting that MET inhibition alone is sufficient to trigger apoptosis. We also quantified apoptosis at 48 h of treatment with inhibitors using Annexin V staining (**Fig. 5D** **and Fig. S3A, B**). There was a four-fold increase in apoptosis detected via Annexin V staining in H2596 cells treated with ARQ 197 that almost doubled with the combinatorial treatment with GDC-0980. This effect was not observed with NVP-BEZ235 (**Fig. 5D**).

### Effect of MET Inhibition on Kinase Profiling using PamGene Microarray

**Figure 6:**
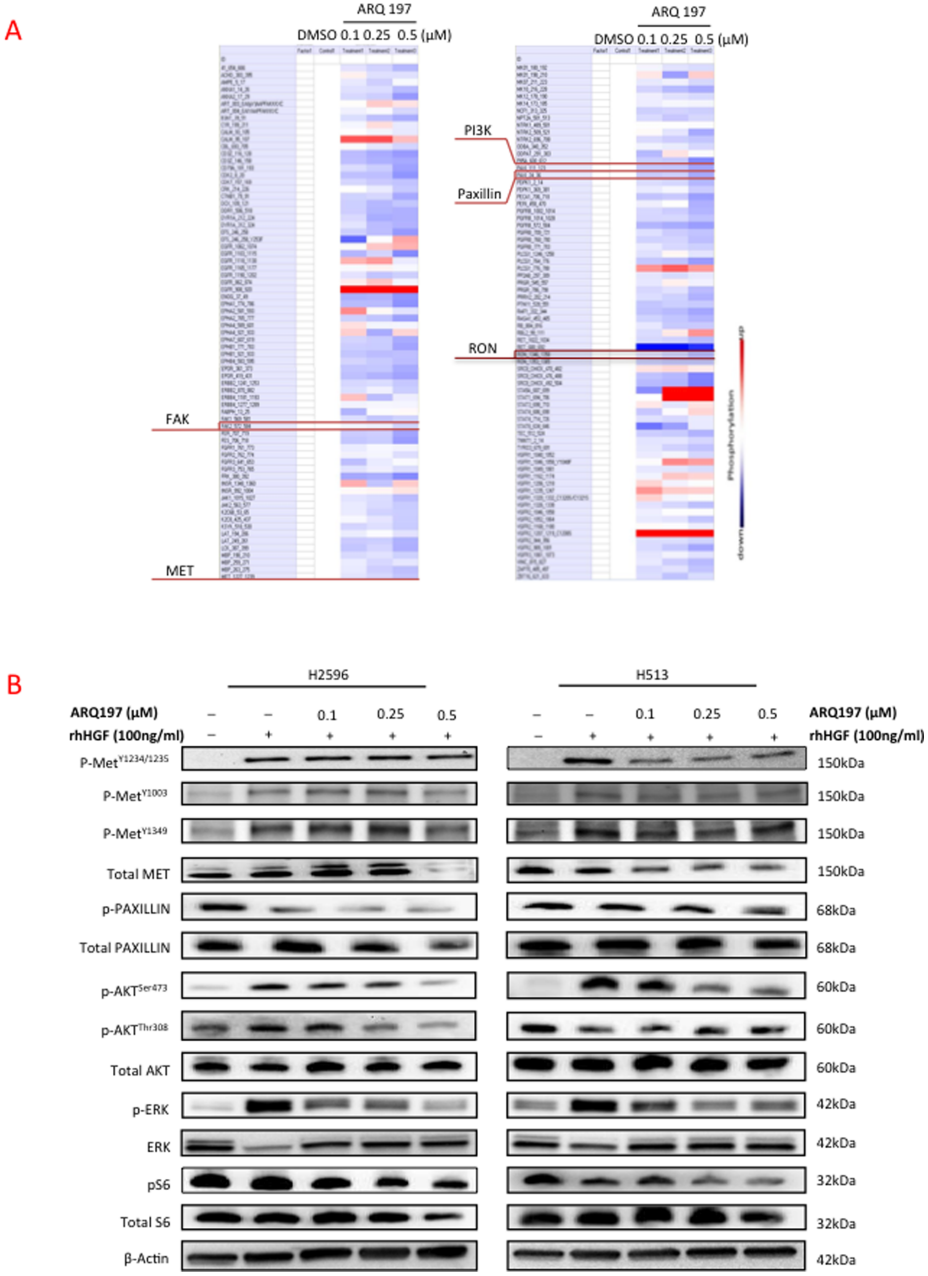
Effect of MET inhibitor ARQ 197 on kinase activation profile and downstream signaling pathways in MPM. The protein tyrosine kinase activity profile obtained using H513 lysates treated with ARQ 197 as tested on PamChip microarrays. The color-coded response signature **(A)** is shown as heatmap in which treatment related upregulation of kinase activity is shown by red and downregulation by blue. Protein tyrosine kinase activity is down regulated in a dose dependent manner for most of the peptides. **(B)** H2596 and H513 cells were starved in 0.5% BSA media for 16 to 18 h and then treated with ARQ 197 at indicated concentrations for 24 h. The cells were then stimulated with human recombinant HGF (100 ng/ml) for 15 min before preparing the cell lysates. Immunobloting was then performed with the following antibodies Phospho-MET^Y1234/1235^, Phospho-MET^Y1003^, Phospho-MET^Y1349^, Phospho-Paxillin, Phospho-AKT ^Ser473^, Phospho -AKT ^thr308^, Phospho-ERK, phospho-S6K and the corresponding total antibodies.

In order to establish that ARQ 197 indeed inhibited MET in MPM cells and to identify other downstream targets, we treated H513 cells with varying concentrations of ARQ 197 and subjected the lysates to PamGene profiling. As expected, there was significant inhibition of MET phosphorylation and surprisingly, RON phosphorylation (**Fig. 6A**). The phosphorylation status of MET downstream targets such as PI3K (p85), FAK and paxillin also showed dose related decreases. To further confirm the inhibition of phosphorylation of MET with treatment of ARQ 197 we treated the H513 and H2596 cells with varying concentrations of ARQ 197 in the presence of HGF and subjected the lysates to immunoblotting (**Fig 6B**). Here we have used three different p-MET antibodies to check the phosphorylation status of MET. With p-MET 1234/1235, there was a clear-cut inhibition in phosphorylation of MET in H513 cells, but less so in H2596 cells. Although with p-MET pY1349 and pY1003 antibodies results are less significant, it showed the same trend in H513 cells. In H2596 cells the inhibition of p-MET was observed at the higher concentration of ARQ 197. MAPK activity is known to mediate cell motility, and promote viability and metastasis. Since ARQ 197 was the main suppressor of MPM cell motility and as combination with PI3K/mTOR inhibitors had no added advantage, we also determined the effect of ARQ 197 on MAPK activity in both H2596 and H513 cells. HGF induced MAPK activity was dramatically suppressed with the treatment of ARQ 197 (**Fig 6B**).

### Effect of Combined Use of MET and PI3K/mTOR Inhibitors on Downstream Signaling Pathways

**Figure 7:**
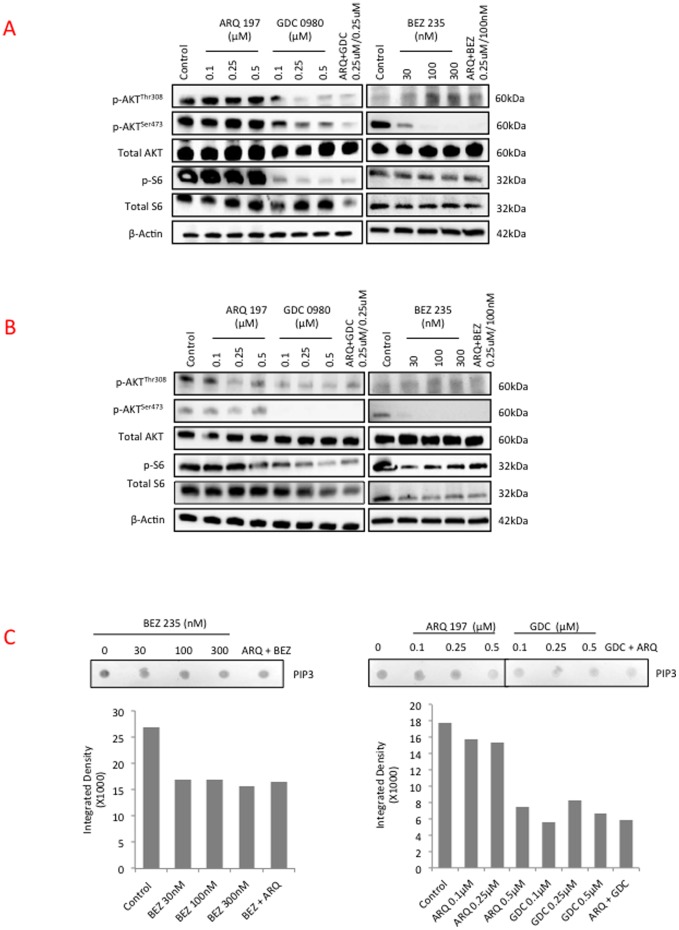
Effect of combined MET and PI3K/mTOR inhibition on kinase activation profile and downstream signaling pathways in MPM. H2596 and H513 cells were plated in 10 cm tissue culture plates overnight and next day treated with indicated inhibitors for 4 h. Phospho-AKT (p-AKT ^Ser473^, p-AKT ^thr308^), total AKT, p-S6K and total S6K were assessed in **(A)** H2596 and **(B)** H513 by immunoblotting. **(C)** H2596 cells were plated in 10 cm tissue culture plates overnight and next day treated with indicated inhibitors for 24 h. PIP3 content was measured via dot blot and densitometric analysis for each blot is shown.

H2596 and H513 cells were treated with increasing concentrations of ARQ 197, or GDC-0980 or NVP-BEZ235 alone or in combination (ARQ 197/GDC-0980 and ARQ 197/NVP-BEZ235) for 4 h. Cell lysates were subjected to immunoblotting and representative blots are shown (**Fig. 7A, B**). While AKT phosphorylation (Ser^473^, Thr308) was not significantly affected by ARQ 197 treatment in either H2596 or H513 cells, the PI3K/mTOR inhibitors NVP-BEZ235 and GDC-0980 had a dramatic suppressive effect (**Fig. 7A, B**). The activation of ribosomal S6 kinase, that is immediately downstream of mTOR, was effectively inhibited by both NVP-BEZ235 and GDC-0980; however ARQ 197 had a suppressive effect only at the highest concentration tested in H513 but not in H2596 cells (**Fig. 7A, B**). Further, we examined the levels of PIP3 the product of p110α in these cells [17]. H2596 cells were incubated with ARQ 197, GDC-0980 or NVP-BEZ235 alone or in combination (ARQ 197/GDC-0980 and ARQ 197/NVP-BEZ235) for 24 h. A representative dot blot and accompanying densitometric analysis is shown (**Fig. 7C**). As expected from the immunoblot results, ARQ 197 had a less dramatic effect on PIP3 production compared to NVP-BEZ235 or GDC-0980. GDC-0980 showed the largest inhibition of PIP3 production, even at low concentration (0.1 µM).

Taken together, the PI3K/mTOR inhibitors, more than ARQ 197 appear to efficiently suppress the growth providing signals mediated through AKT and S6 kinase while ARQ 197 suppressed cell motility and induced apoptosis.

### Anti-MM Tumor Activity of MET and PI3K/mTOR Inhibitors in a Mouse Xenograft Model

**Figure 8:**
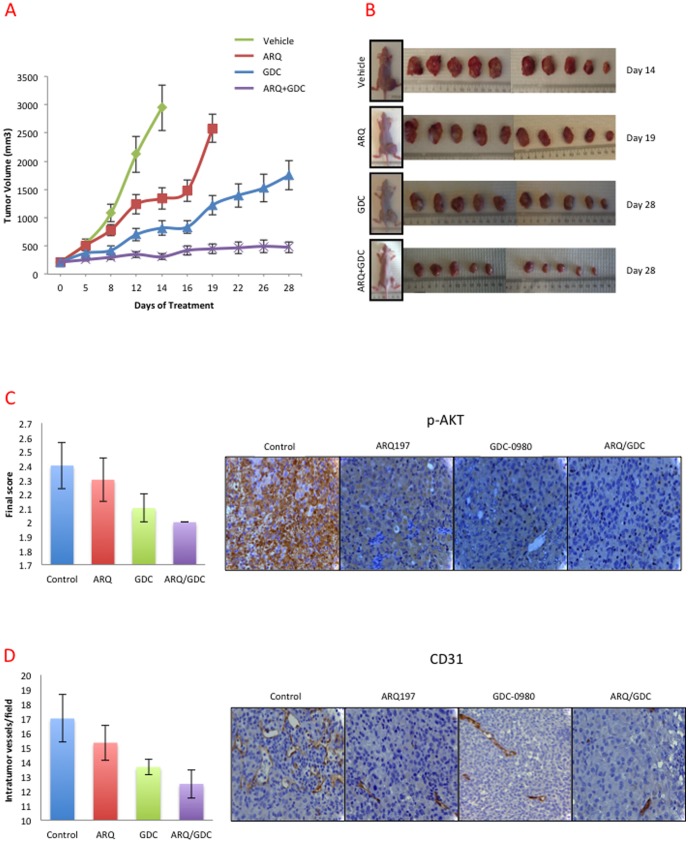
ARQ 197 and GDC-0980 inhibit the growth of tumor xenografts in nude mice and their combination has synergistic inhibition of tumor growth. Results from tumor xenograft experiments testing the efficacy of ARQ 197 and GDC-0980 alone and/or combination in inhibiting the growth of H2596 cell line tumors in nude mice. Oral gavage treatment with ARQ 197 (200 mg/kg/day) and/or GDC-0980 (5 mg/kg/day) reduced H2596 tumor growth significantly relative to vehicle control (p<0.001). The combined use of ARQ 197/GDC-0980 was much more effective than any single agent alone, in inhibiting the tumor growth (p<0.01). **(A)** Tumor sizes were recorded every three days till the end of the experiment. **(B)** Representative images of mice and tumors treated with vehicle control, ARQ 197, GDC-0980 and their combination. **(C)** Immunohistochemical staining of tumor tissues with total CD31 and p-AKT and quantification. Bar graphs indicate average expression with standard error.

The antitumor activity of MET inhibitor ARQ 197 and PI3K/mTOR inhibitor alone or in combination was further investigated in a mouse xenograft model derived from H2596 cells. Mice were treated daily by oral gavage with vehicle, ARQ 197, GDC-0980 or their combination. The oral regimen started on 22nd day of MPM cell xenograft, when tumors reached an average volume of 200 mm^3^. Although treatment with ARQ 197 or GDC-0980 alone inhibited tumor growth, the effect of GDC-0980 was far greater than ARQ 197 (**Fig. 8A, B**). The vehicle control and ARQ 197 group mice were sacrificed on day 19 due to higher tumor burden and morbidity. Mice treated with GDC-0980 alone or ARQ 197/GDC-0980 were sacrificed on day 28. The combination of ARQ 197/GDC-0980 had a more significant effect on suppression of tumor growth compared to any single drug (p<0.001). The average tumor volume was less in mice treated with ARQ 197/GDC-0980, followed GDC-0980 and ARQ 197 groups (**Fig. 8A, B**). The ARQ 197/GDC-0980 treated mice did not suffer any body weight reduction, suggesting that the combination of these two drugs was well tolerated (**Fig. S4**).

Tumor sections from vehicle control, ARQ 197, GDC-0980 and combination treated mice were immunostained with p-MET, total MET, CD31 and p-AKT. We did not observe any significant change in the expression of total MET and p-MET in all the groups (data not shown). However there was a significant decrease in p-AKT which is a key downstream signaling molecule of PI3K and MET (**Fig. 8C**). CD31, an indicator of angiogenesis was also decreased in the both MET and PI3K/mTOR inhibitor treated mice as compared to vehicle treated mice (**Fig. 8D**).

## Discussion

MPM is highly resistant to radiation therapy and the preferred treatments are surgery (at early stages) and a combinatorial treatment with cisplatin and pemetrexed [6], [7]. The average survival time after diagnosis is one year highlighting the pressing need for the development of more effective therapies. Here we have clearly demonstrated the benefit of combined targeting of the RTK, MET and the key intracellular signal transducer, PI3K, using *in vitro* and *in vivo* MPM xenograft models. As expected, the MET inhibitor ARQ 197 and the PI3K/mTOR inhibitors GDC-0980 and NVP-BEZ235 when used alone significantly decreased MPM cell viability (**Fig. 2A–D**); however only ARQ 197 adversely affected the cell motility thereby indicating that HGF/MET signaling promotes MPM cell motility independent of the PI3K/mTOR pathway (**Fig. 4A–D**). The combination of ARQ 197 with either GDC-0980 or NVP-BEZ235 had a strong synergistic suppressive effect on MPM cell viability (**Fig. 3A-D**). The underlying mechanism involved cell cycle arrest and induction of apoptosis. While ARQ 197 induced cell cycle arrest at G2/M phase, the PI3K/mTOR inhibitors induced G0/G1 arrest; the combination mainly caused accumulation of MPM cells at G2/M. The MET inhibitor was a strong inducer of apoptosis in MPM cells. ARQ 197, exhibited strong inhibition of MET autophosphorylation (Y1234-1235) in H513 cells, but not in H2596 cells. With two other p-MET antibodies the same trend was found in H513 cells, but in the case of H2596 cells p-MET inhibition was observed only at high doses. (**Fig. 6** **B**). ARQ 197 also had a strong suppressive effect on HGF induced MAPK activation (**Fig. 6** **B**). The majority of the inhibitory effect on downstream AKT and S6 kinases could be mostly attributed to the two PI3K/mTOR inhibitors used. These findings were substantiated by PamGene microarray analysis using ARQ 197. These results showed that MET, as well as the related RON kinase, were inhibited by ARQ 197 treatment. Also, the phosphorylation of downstream targets such as the p85 subunit of PI3K, FAK and paxillin were dampened. In addition, PI3K/mTOR inhibitors were more effective in suppressing the phosphorylation of AKT and S6 kinases and production of PIP3 compared to the MET inhibitor. Finally, using a xenograft model, we demonstrated a highly synergetic effect of combinatorial use of ARQ 197 with GDC-0980 in the suppression of MPM tumor growth.

We previously showed that MET is overexpressed and active in most of the MPM cell lines tested as well as in archival MPM tissues. Here, we have shown that key MET downstream signaling molecules including PI3K (p85 and p110α subunits), AKT and phospho-AKT are also expressed in majority of the cell lines tested. The archival human MPM tissue samples revealed significant overexpression of p-AKT, an indication of activated PI3K, a common feature shared by a variety of cancers [31]–[34].

In addition to overexpression of MET in MPM, we previously identified mutations within the MET semaphorin domain (N375S, M431V, and N454I), the juxtamembrane domain (T1010I and G1085X), and an alternative spliced form exon 10 deletion. The cell lines H513 and H2596 harbor the T1010I mutation and exhibited the most dramatic reduction of cell growth when treated with the MET inhibitor SU11274 [13]. Here we tested ARQ 197, a non-ATP competitor of MET tyrosine kinase activity, reported to suppress the growth of multiple cancer cell lines [16]. Cell viability was decreased in all mesothelioma cell lines tested in response to ARQ 197, with those harboring the T1010I MET mutation (H513 and H2596) showing the greatest sensitivity.

The ability of ARQ 197 to specifically inhibit MET kinase activity is controversial. Previous reports on ARQ 197 have shown it to predominantly inhibit MET but not its related kinase RON [16]. *In vitro* studies using purified MET indicated that ARQ 197 appeared to only bind to non-phosphorylated MET kinase domain and prevented autophosphorylation in a time dependent manner [35]. More recently other reports have emerged to challenge this view. Studies using multiple cancer cell types have failed to detect any inhibition of HGF-induced MET phosphorylation in ARQ 197 treated cells [36], [37] although cell growth was still inhibited. ARQ 197 was observed to disrupt microtubule assembly and induce cell cycle arrest in these cells [36], [37]. Unlike crizotinib and PHA 665792, two MET ATP-competitive inhibitors, ARQ 197 inhibited the growth of both MET addicted and non-addicted cancer cell lines [37]. Our studies using PamGene microarray analysis revealed that ARQ 197 indeed targets MET in mesothelioma cells; however it also inhibited RON (**Fig. 6A**). The immunoblot analysis of the H513 and H2596 cells pretreated with ARQ 197 and stimulated with HGF showed significant inhibition of p-MET (1234/1235) in H513 cells, but less so in H2596. Using other p-MET antibodies (pY1349 and pY1003) it showed the same trend in H513 cells, and in H2596 it inhibited phosphorylation of MET at higher concentration. In our hands, ARQ 197 appeared to have no significant effect on EGFR activity and EGFR is known to be overexpressed in MPM [38] (data not shown). EPHB4, another prominent RTK expressed in MPM [39] also appeared to be inhibited. FAK is a prominent downstream kinase in MET signaling [40] and it's phosphorylation is suppressed by ARQ 197 in a dose dependent fashion (**Fig. 6A**). Moreover, our data on the effect of ARQ 197 on cell viability is in agreement with our previous report on the effect of SU11274, another MET kinase inhibitor, on MPM cell viability and growth (4). In addition, that study also demonstrated that MET knockdown using specific siRNA in MPM cells induced significant apoptosis that mirrors our present finding that ARQ 197 also induces apoptosis in MPM cells (**Figure 5B–D**). It is therefore our contention that the effects of ARQ 197 seen on MPM cells are mainly mediated through suppression of MET activity.

We have previously shown that the cytoskeletal adaptor protein paxillin, a key MET downstream signaling molecule, plays a significant positive role in lung tumor development [41]. Our studies show that ARQ 197 inhibited phosphorylation of paxillin thereby attesting to the ability of ARQ 197 to adversely affect the cytoskeleton either through MET dependent or independent pathways. Apart from paxillin, which mediates directional cell motility, MAPK, a key intracellular signaling mediator and transcription factor is also known to promote cell motility [42], [43]. Here we also demonstrated that treatment of H2596 and H513 MPM cells had a dramatic inhibitory effect on suppression of HGF induced MAPK and paxillin phosphorylation thereby explaining the underlying mechanism in ARQ 197 mediated suppression of cell motility seen in the above two MPM cell lines.

PI3K signals impact cancer cell growth, survival, motility, and the p110α is known to acquire gain-of-function mutations in several cancers [44]. However, in the mesothelioma genomic DNA extracts tested here, we did not detect any PI3K mutations (data not shown). mTOR is a conserved PI3K-related ser/thr kinase that plays a pivotal role in tumor growth as it funnels signals from both receptors and nutrients to activate cell growth [45]. It is upregulated in various cancers and may have a role in mediating resistance to EGFR tyrosine kinase inhibition [46], [47]. Our results from the use of PI3K/mTOR dual inhibitor in MPM cells clearly support the above contention and the downstream connections between PI3K, AKT, mTOR and the ribosomal S6 kinase. The ribosomal S6 kinase is placed downstream of mTOR. mTORC1 when activated induces phosphorylation of ribosomal S6 kinases that then phosphorylates rS5. Due to the negative feedback loop between S6K and IRS, simultaneously targeting PI3K and mTOR kinases is a better approach than using inhibitors that target either PI3K or mTOR alone. In the present study, we initially determined the efficacy of PI3K first generation and second-generation inhibitors such as wortmanin, LY294002 and GDC-0941to curb MPM cell growth without much success (data not shown) and therefore shifted the focus to PI3K/mTOR dual inhibitors. GDC-0980 was reported to be a potent inhibitor of breast, prostate and lung cancer cell growth and was much less effective against melanoma and pancreatic cancers, most likely due to the presence of highly active KRAS and BRAF usually encountered in these cancers. GDC-0980 was shown to induce G1 cell cycle arrest followed by apoptosis in certain cancer cell lines. Relatively low doses also inhibited xenograft tumor growth [23]. Both PI3K and mTOR dual inhibitors used here induced MPM cells to accumulate in G0/G1 phase (**Fig. 5A**); and were not very efficient at inducing PARP cleavage, a marker of apoptosis (**Fig. 5B, C**). They were however, formidable inhibitors of *in vitro* MPM cell growth and *in vivo* MPM xenograft tumor growth in the nude mouse when combined with ARQ 197 (**Fig. 8A, B**). NVP-BEZ235 yielded promising results against variety of cancers [21], [48]–[51]. It induced growth arrest in renal cell carcinoma cells both *in vitro* and *in vivo* and was more effective compared to Rapamycin, a TORC1 (mTOR) inhibitor [21]. Using glioma cells, it was shown that NVP-BEZ235 specifically inhibited PI3K/mTOR signaling; an observation supported by suppressed activity of AKT, and S6K1. The cells also underwent autophagy and cell cycle arrest at G0/G1 phase. In addition, there was a significant anti-angiogenic effect as evidenced in mouse xenograft studies [50]. The present studies are in complete accordance with the above findings. For instance, both NVP-BEZ235 and GDC-0980 effectively suppressed AKT and S6K phosphorylation and production of PIP3 in a dose dependent manner (**Fig. 7** **A, B, C**) and arrested MPM cells in G0/G1 phase (**Fig. 5A** **, and Fig. S1A, B**).

It has been observed that inhibition of mTOR results in enhanced cancer cell death [52]. In glioma cells, in addition to inducing G0/G1 cell cycle arrest, NVP-BEZ235 was also observed to enhance autophagy [51]. The efficacy of NVP-BEZ235 in suppressing lung cancer cell growth was significantly enhanced by combining it with chloroquine, an inhibitor of autophagy [51]. MET inhibition was also observed to enhance autophagy in gastric adenoma cells [53]. In our hands, NVP-BEZ235 showed significant synergy in killing MPM cells when combined with ARQ 197 (**Fig. 3A, B**); however, we have not checked the status of autophagy in these cells. GDC-0980 also showed significant synergy in combination with ARQ 197 in MPM (**Fig. 3C, D**).

Several MET inhibitors are in various phases of cancer clinical trials [54]; however no studies to date have been reported using a combination of MET and PI3K inhibitors, especially in MPM. A recent investigation reported significant synergistic inhibitory effect on MPM cell growth with combined treatment of MEK inhibitor U0126 and the PI3K inhibitor LY 294002 *in vitro* and *in vivo* mouse models [33]. Based on our previous study and the data presented here, we have shown that both MET and PI3K are overexpressed in MPM [13], [34]. Unlike other cancers [44], MPM does not appear to undergo frequent gain of function mutations in p110α (data not shown), however IHC analysis of archival MPM tumor samples revealed overexpression of p-AKT that supports the presence of highly active PI3K (**Fig. 1A, B**).

In summary, using MPM cell lines and a mouse xenograft model, we have clearly demonstrated the advantage of the combinatorial targeting of MET and PI3K/mTOR in MPM. PI3K is a key downstream signaling molecule in the HGF/MET pathway and significant synergy was observed when ARQ 197 was combined with either GDC-0980 or NVP-BEZ235 in suppressing MPM cell motility and growth and *in vivo* tumor development. The observed synergy could be due to the fact that several RTKs, apart from MET, are likely to be active in MPM that all feed into the PI3K/mTOR pathway. Therefore, dual targeting MET and PI3K/mTOR is logical. Our mouse study revealed a dramatic suppressive effect of ARQ 197/GDC-0980 on *in vivo* MPM tumor growth that is promising and warrants further translational studies.

## Supporting Information

Figure S1 **MET inhibitor alone or in combination with PI3K/mTOR dual inhibitors induces cell cycle arrest.** H2596 cells were treated with ARQ 197(0.2 µM), GDC-0980 (0.2 µM), NVP-BEZ235 (60 nM) alone and in combination for 48 h. Cell cycle profile was determined using flow cytometry after staining with PI/RNase, representative flow cytometry profiles are shown in **(A)**. The percentages of cells in G1, S, and G2/M phases were quantified and the results expressed as the mean ± SEM of four independent experiments as shown in **(B)**.
(TIF)

Figure S2 **Effect of ARQ 197(MET inhibitor), GDC-0980, BEZ 235 (PI3K/mTOR inhibitor) alone and in combination on cleaved PARP (Marker of apoptosis) in H2596 cells.** H2596 cells were treated with ARQ 197(0.2 µM), GDC-0980 (0.2 µM), NVP-BEZ235 (60 nM) alone and in combination for 48 h. Cell were then fixed in 4% paraformaldehyde and stained for cleaved PARP and actin as described in Methods S1.
(TIF)

Figure S3 **Effect of ARQ 197, GDC-0980, NVP-BEZ235 alone and in combination on apoptosis of H2596 Cells.** H2596 cells treated with ARQ 197(0.2 µM), GDC-0980 (0.2 µM), NVP-BEZ235 (60 nM) alone and in combination for 48 h as indicated, the cells were then stained with Annexin V-FITC/PI and analyzed by flow cytometry. Representative flow cytometry profiles are shown **(A)**. Results are expressed as mean percentage of apoptotic cells ± SEM of four independent experiments **(B)**.
(TIF)

Figure S4 **Mouse body weight during H2596 xenograft and drug treatment.** Mice were injected with H2596 cells on the right flank and tumor growth was followed until the 22nd day of MPM cell xenograft, when tumors reached an average volume of 200 mm^3^. Mice were then treated daily by oral gavage with vehicle, ARQ 197, GDC-0980 or their combination and mouse body weight was recorded every three days.
(TIF)

Methods S1 **Immunofluorescence and Confocal Microscopy.**
(DOCX)
