# Supplementary figures and images for: MET and PI3K/mTOR as a Potential Combinatorial Therapeutic Target in Malignant Pleural Mesothelioma
Source: PLoS One. 2014 Sep 15;9(9):e105919. doi: 10.1371/journal.pone.0105919 (PMC4164360; doi:10.1371/journal.pone.0105919)

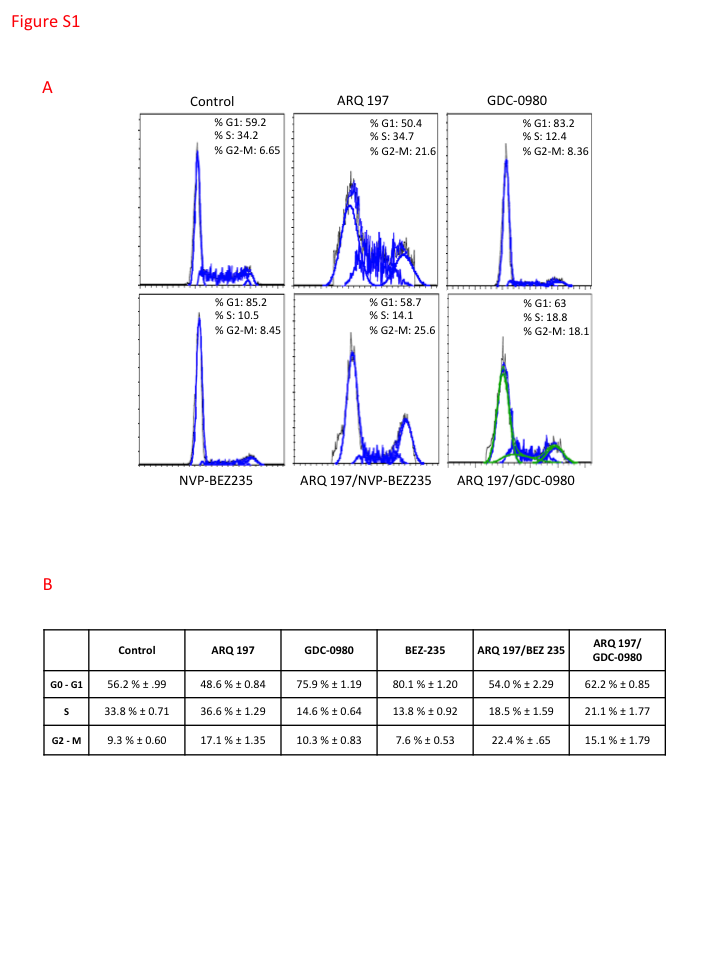

Supplement: Figure S1 — MET inhibitor alone or in combination with PI3K/mTOR dual inhibitors induces cell cycle arrest. H2596 cells were treated with ARQ 197(0.2 µM), GDC-0980 (0.2 µM), NVP-BEZ235 (60 nM) alone and in combination for 48 h. Cell cycle profile was determined using flow cytometry after staining with PI/RNase, representative flow cytometry profiles are shown in (A). The percentages of cells in G1, S, and G2/M phases were quantified and the results expressed as the mean ± SEM of four independent experiments as shown in (B). (TIF) [file pone.0105919.s001.tif]

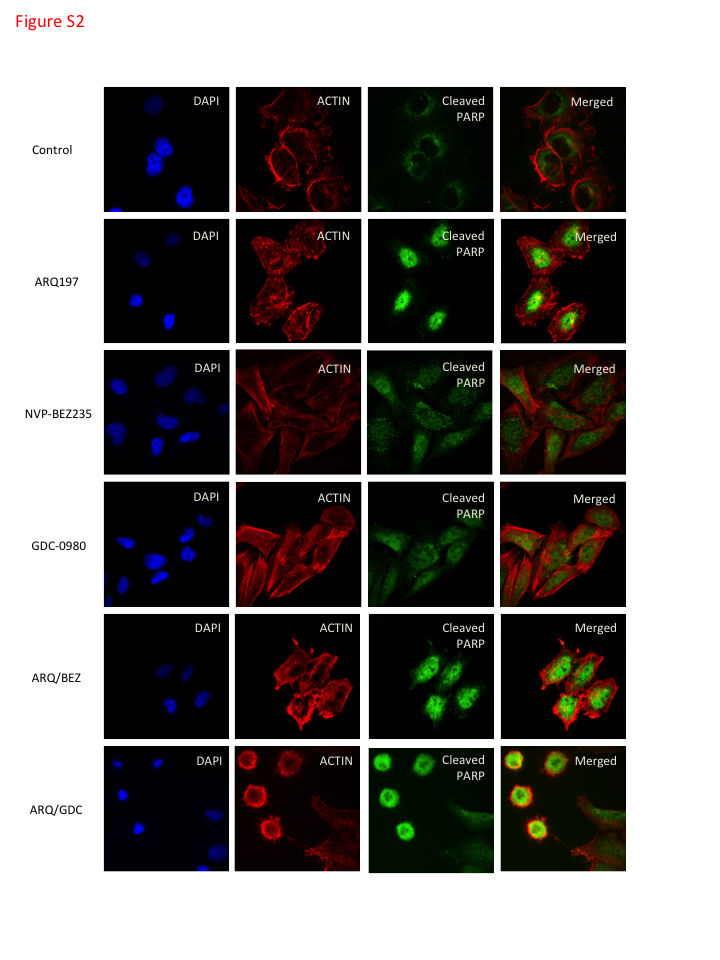

Supplement: Figure S2 — Effect of ARQ 197(MET inhibitor), GDC-0980, BEZ 235 (PI3K/mTOR inhibitor) alone and in combination on cleaved PARP (Marker of apoptosis) in H2596 cells. H2596 cells were treated with ARQ 197(0.2 µM), GDC-0980 (0.2 µM), NVP-BEZ235 (60 nM) alone and in combination for 48 h. Cell were then fixed in 4% paraformaldehyde and stained for cleaved PARP and actin as described in Methods S1. (TIF) [file pone.0105919.s002.tif]

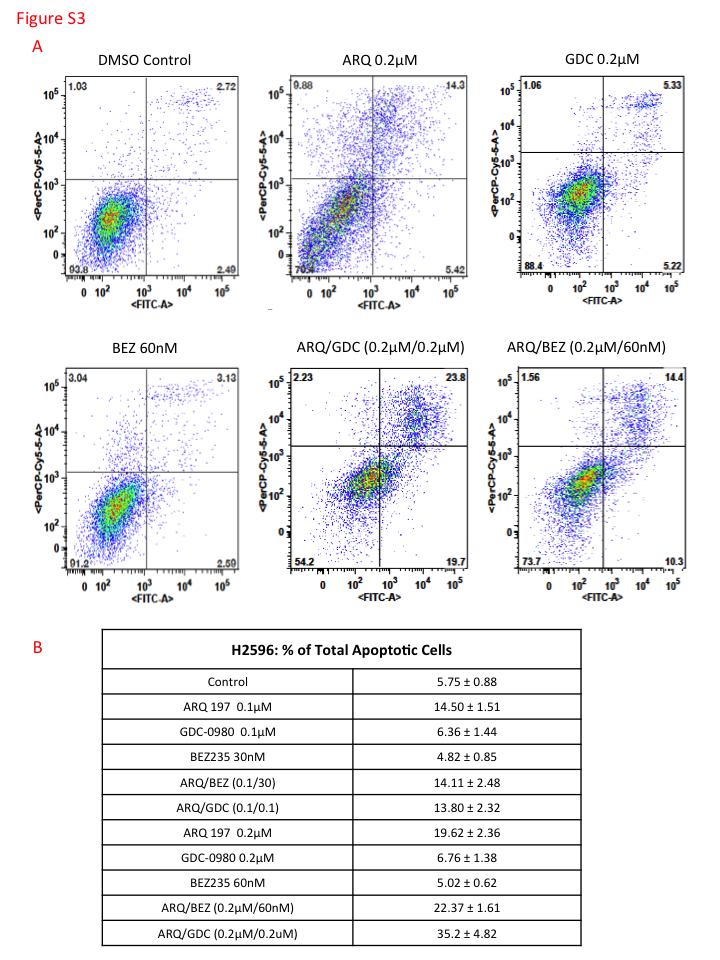

Supplement: Figure S3 — Effect of ARQ 197, GDC-0980, NVP-BEZ235 alone and in combination on apoptosis of H2596 Cells. H2596 cells treated with ARQ 197(0.2 µM), GDC-0980 (0.2 µM), NVP-BEZ235 (60 nM) alone and in combination for 48 h as indicated, the cells were then stained with Annexin V-FITC/PI and analyzed by flow cytometry. Representative flow cytometry profiles are shown (A). Results are expressed as mean percentage of apoptotic cells ± SEM of four independent experiments (B). (TIF) [file pone.0105919.s003.tif]

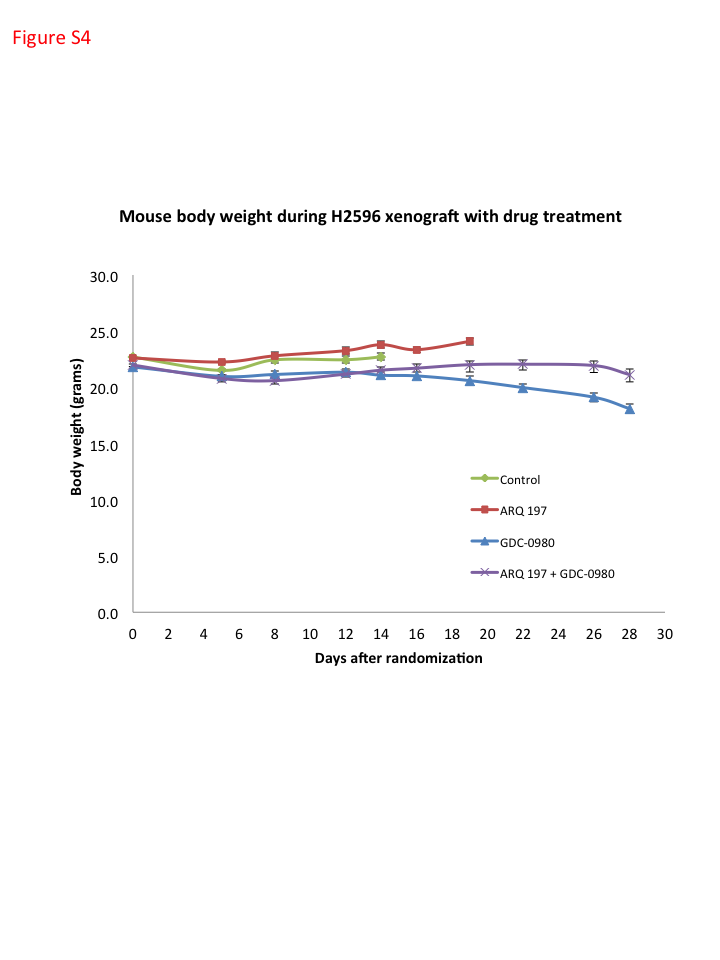

Supplement: Figure S4 — Mouse body weight during H2596 xenograft and drug treatment. Mice were injected with H2596 cells on the right flank and tumor growth was followed until the 22nd day of MPM cell xenograft, when tumors reached an average volume of 200 mm3. Mice were then treated daily by oral gavage with vehicle, ARQ 197, GDC-0980 or their combination and mouse body weight was recorded every three days. (TIF) [file pone.0105919.s004.tif]
