# Supplementary material for: MET and PI3K/mTOR as a Potential Combinatorial Therapeutic Target in Malignant Pleural Mesothelioma
Source: PLoS One. 2014 Sep 15;9(9):e105919. doi: 10.1371/journal.pone.0105919 (PMC4164360; doi:10.1371/journal.pone.0105919)
Supplement: Methods S1 — Immunofluorescence and Confocal Microscopy. (DOCX) [file pone.0105919.s005.docx]

**Supplemental Materials and Methods**

**Immunofluorescence and Confocal Microscopy:**

Cells were grown in 10% FBS media on glass coverslips in 6 well tissue culture plates overnight. Next day they were treated with indicated concentration of ARQ 197, NVP-BEZ235, GDC-0980 individually or in combination (ARQ 197/NVP-BEZ235 and ARQ 197/GDC-0980) for 6 h. The cells were then washed with PBS and then fixed with 4% paraformaldehyde for 15 min at RT. Fixed cells were washed twice with ice-cold PBS and then permeabilized with 0.1% Triton X 100 in PBS for 5 min. The cells were then blocked in 10% BSA in PBS for 1h at RT. They were then incubated with the appropriate primary antibody in 2.5% BSA for 1 h followed by three washes with PBS. The cells were then incubated with appropriate fluorescent-labeled secondary antibodies for 1h. After washing three times with PBS, the coverslips were mounted on slides using Vectashield. The fluorescent pictures were visualized and captured using microscope and imager.

**Supplementary Figure legends**

**Supplemental Figure 1: MET inhibitor alone or in combination with PI3K/mTOR dual inhibitors induces cell cycle arrest.**

H2596 cells were treated with ARQ 197(0.2μM), GDC-0980 (0.2μM), NVP-BEZ235 (60nM) alone and in combination for 48 h. Cell cycle profile was determined using flow cytometry after staining with PI/RNase, representative flow cytometry profiles are shown in **(A)**. The percentages of cells in G1, S, and G2/M phases was quantified and the results expressed as the mean ± SEM of four independent experiments as shown in **(B).**

**Supplemental Figure 2:** **Effect of ARQ 197(MET inhibitor), GDC-0980, BEZ 235 (PI3K/mTOR inhibitor) alone and in combination on cleaved PARP (Marker of apoptosis) in H2596 cells.**

H2596 cells were treated with ARQ 197(0.2μM), GDC-0980 (0.2μM), NVP-BEZ235 (60nM) alone and in combination for 48 h. Cell were then fixed in 4% paraformaldehyde and stained for cleaved PARP and actin as indicated.

**Supplemental Figure 3: Effect of ARQ 197, GDC-0980, NVP-BEZ235 alone and in combination on apoptosis of H2596 Cells.**

H2596 cells treated with ARQ 197(0.2μM), GDC-0980 (0.2μM), NVP-BEZ235 (60nM) alone and in combination for 48 h as indicated, the cells were then stained with Annexin V-FITC/PI and analyzed by flow cytometry. Representative flow cytometry profiles are shown **(A).** Results are expressed as mean percentage of apoptotic cells ± SEM of four independent experiments **(B)**.

**Supplemental Figure 4: Mouse body weight during H2596 xenograft and drug treatment.**

Mice were injected with H2596 cells on the right flank and tumor growth was followed until the 22nd day of MPM cell xenograft, when tumors reached an average volume of 200 mm^3^. Mice were then treated daily by oral gavage with vehicle, ARQ 197, GDC-0980 or their combination and mouse body weight was recorded every three days.
